# The Oncology Research Information Exchange Network (ORIEN) - Building a Real-World Collaborative, Patient-driven Infrastructure for Discovery Research and Precision Oncology

**DOI:** 10.21203/rs.3.rs-7077810/v1

**Published:** 2026-06-02

**Authors:** Michelle L. Churchman, Xin Lu, David M. McKean, Michael D. Radmacher, Qi Zhang, Robert J. Rounbehler, Aik Choon Tan, Timothy I Shaw, Alyssa Obermayer, G. Daniel Grass, Daniel Spakowicz, Ahmad A. Tarhini, Kenneth H. Shain, Rafael Renatino Canevarolo, Mark B. Meads, Ariosto S. Silva, Praneeth Reddy Sudalagunta, Brandon J. Manley, Shane Huntsman, Deborah Collyar, Jill M. Kolesar, B. Mark Evers, Nancy Single, Stephen B. Edge, Candace S. Johnson, George J. Weiner, Martin D. McCarter, Virginia F. Borges, Latrisha Horne, James W. Lillard, Dinesh Pal Mudaranthakam, Julia White, Steven Libutti, Shridar Ganesan, Gregory M. Riedlinger, Thomas P. Loughran, Dina Gould Halme, Margaret A. Johns, Cletus Arciero, Bodour Salhia, Abdul Rafeh Naqash, Craig D. Shriver, Kelvin P. Lee, Bryan P. Schneider, David A. Nix, Gary C. Fillmore, Howard Colman, Cornelia M. Ulrich, Brian C. Springer, Raphael Pollock, Emily A. Vucic, Jeff Sherman, Anshu K. Jain, William S. Dalton, Phaedra Agius, Erin M. Siegel

**Affiliations:** 1 Aster Insights, Hudson, FL 34667, USA; 2 Huntsman Cancer Institute, University of Utah, Salt Lake City, UT 84132, USA; 3 H. Lee Moffitt Cancer Center and Research Institute, Tampa, FL 33612, USA; 4 The Ohio State University Comprehensive Cancer Center, Columbus, OH 43210, USA; 5 Patient Advocates in Research, Danville, California 94526, USA; 6 University of Kentucky Markey Cancer Center, Lexington, KY 40536, USA; 7 Roswell Park Comprehensive Cancer Institute, Buffalo, NY 14263, USA; 8 University of Iowa Holden Comprehensive Cancer Center, Iowa City, IA 52242, USA; 9 University of Colorado Cancer Center, Aurora, CO, USA; 10 Morehouse School of Medicine, Atlanta, GA 30310 USA; 11 University of Kansas Comprehensive Cancer Center, Kansas City, KS 66103, USA; 12 Rutgers Cancer Institute, New Brunswick, NJ 08901, USA; 13 University of Virginia Cancer Center, Charlottesville, VA 22903, USA; 14 Winship Cancer Institute of Emory University, Atlanta, GA 30322, USA; 15 USC Norris Comprehensive Cancer Center, Los Angeles, CA 90033, USA; 16 Oklahoma University Health Stephenson Cancer Center, Oklahoma City, OK 73104, USA; 17 Murtha Cancer Center, Uniformed Services University, Bethesda, MD 20889, USA; 18 Indiana University Simon Comprehensive Cancer Center, Indianapolis, IN 46202, USA; 19 Zephyr AI, Washington, DC, USA

## Abstract

Over a decade ago, collaborating academic cancer centers formed the Oncology Research Information Exchange Network^®^ (ORIEN) to develop a patient-driven, federated infrastructure for oncology research. Aster Insights is the network’s operational, commercial, and research partner. Together ORIEN and Aster Insights have built a unique multimodal dataset on the active engagement and consent of patients who opted into the Total Cancer Care^®^ (TCC) protocol to contribute their data and biospecimens for research. Over 400,000 cancer patients have been enrolled in TCC, >32,500 of which have an in silico “Avatar” generated to represent their individual patient experience and molecular profile to support a broad range of network and industry research use cases. We provide an overview of ORIEN’s evolution, demonstrate the power of our data resources through a landmark analysis of >37,000 tumors across all cancer types collected for the Avatar program, and provide a vision for ORIEN to fuel collaborative research.

## INTRODUCTION

Advances in cancer genomics and molecular profiling have transformed our understanding of tumor biology and opened new frontiers in precision oncology.^1,2^ While there are promising success stories of precision oncology, the translation of these discoveries into improved patient outcomes has been limited by the lack of large-scale, harmonized clinicogenomic datasets that integrate longitudinal clinical data with comprehensive molecular and genomic profiles.^3–5^ To address this gap, a national network of collaborating research and clinical centers came together within the Oncology Research Information Exchange Network (ORIEN) in 2014 to develop a uniquely patient-driven, federated infrastructure for discovery research and precision oncology research.^6,7^

Unlike many existing datasets derived from retrospective clinical sequencing or electronic health records,^8 9^ ORIEN has built a clinicogenomic dataset on the prospective engagement and consent of patients who opted into the Total Cancer Care (TCC) protocol^10^ to contribute their data and biospecimens to advance cancer research. ORIEN’s collective impact is further enabled by the efficiencies of master agreements across centers, standardized data model and quality standards, patient advocacy, and multidisciplinary exchange of ideas. ORIEN has a strong partnership with Aster Insights (formerly M2GEN), which provides commercial, operational, and research support to the network.

This report provides an overview of the growth of the ORIEN network, from its initial vision to today, and its signature Avatar initiative to create a uniform interoperable clinicogenomic dataset composed of in-depth abstracted clinical data paired with whole exome and transcriptome sequencing data. We present here a landmark description of over 37,000 tumors from more than 32,500 cancer patients, showcasing the unprecedented breadth and depth of this resource, and highlight use cases that demonstrate its value for research and clinical applications. We also demonstrate the power of ORIEN in creating a collective impact for advancing cancer research through academic collaborations within ORIEN and with industry partners to support in-depth discovery and translational research that will lead to improved patient outcomes.

## RESULTS

### ORIEN - Building a collaborative, patient-driven infrastructure for cancer discovery research and precision oncology

**Figure 1.**
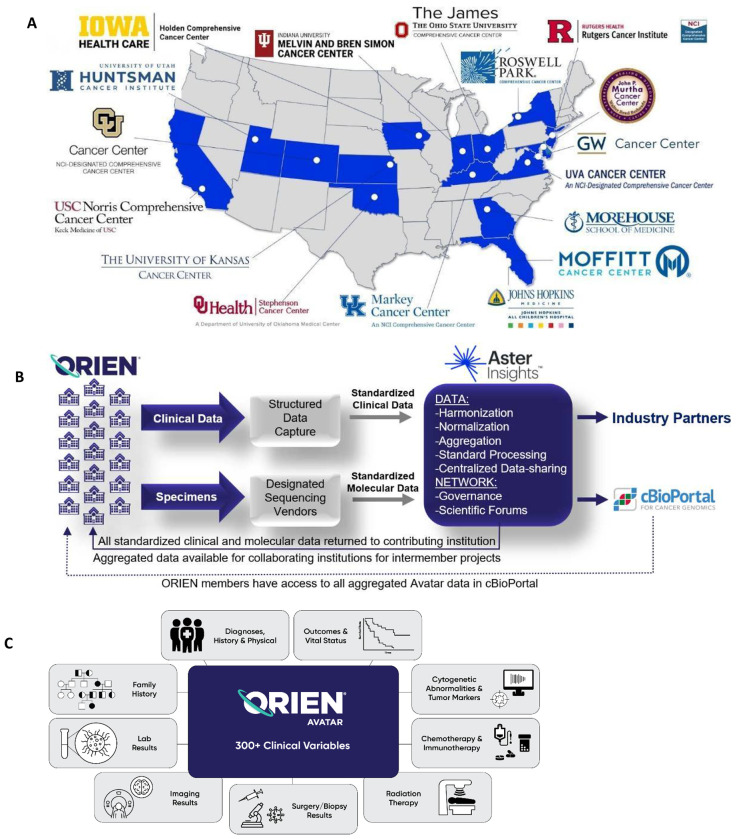
ORIEN Member Map and Model. **A.** Map of the current ORIEN cancer centers across the U.S. **B.** Workflow of ORIEN and Aster Insights partnership and data generation for Avatar Program. **C.** Avatar dataset is a rich longitudinal clinical data comprised of 325 variables.

ORIEN was initially established between two centers, Moffitt Cancer Center and the Ohio State University James Comprehensive Cancer Center,^6,7^ in 2014 and grew quickly to include 19 cancer centers across the U.S. at its peak. There are 17 centers currently participating in ORIEN (Figure 1A). Every member commits to implementing a protocol that includes standard elements of the TCC protocol, including patient consent to contribute and share clinical data and the collection of biospecimens following standard operating procedures.^10^ In addition to TCC, membership comes with an expectation of participation in ORIEN governance, data sharing, and active engagement in collaborative research projects across the network. The guiding principles of inclusiveness of U.S. cancer centers, harmonization of data within a centralized data warehouse, accessibility of data for collaborative use, engaging patients as active partners, and achieving long-term sustainability through public-private partnerships remain at the forefront of the network’s activities.

ORIEN is founded on an ecosystem of shared values^11^ where researchers, patients, industry sponsors, and a data science company are all essential contributors. For patients, ORIEN is guided by patient choice to “opt-in” through active consent to the TCC observational research study and actively engages patients at all levels of network governance through the ORIEN Patient Advisory Council (OPAC). Industry sponsors also contribute to the ecosystem through sponsored research agreements with Aster Insights to fund data generation or to work directly with ORIEN researchers. One way ORIEN is engaging industry sponsors is through the centrally coordinated ORIEN Clinical Trial Network (OCTN), that includes a first-in-kind NCI-approved centralized Protocol Review & Monitoring System (PRMS) scientific review committee (hosted by the Moffitt Cancer Center as part of its Cancer Center Support Grant), master agreements, and a dedicated clinical trial subcommittee. Aster Insights fulfills the critical role as the coordinating body, serving as the central hub supporting governance, collaboration, and developing a path of sustainability through industry partnerships that provide funding for ORIEN initiatives.

### ORIEN’s Avatar Initiative - Creating a Uniform Clinicogenomic Resource

Together ORIEN and Aster Insights have generated a unique clinicogenomic resource that was intentionally designed to support basic, translational, and clinical research in oncology, as well as the drug development lifecycle for industry partners. The Avatar program generates research grade whole-exome sequencing (WES) on germline and tumor specimens, whole transcriptome tumor sequencing (RNASeq), and collects deep longitudinal clinical data (Figure 1B), from a subset of the over 400,000 patients enrolled in the TCC protocol. All 325 abstracted clinical data elements (Figure 1C) and molecular sequencing files are harmonized into a standardized, structured format to enable aggregation of de-identified data for seamless data-sharing via a centralized controlled-access cloud-based platform and ORIEN-specific instance of cBioPortal^12,13^ for data visualization.

**Figure 2.**
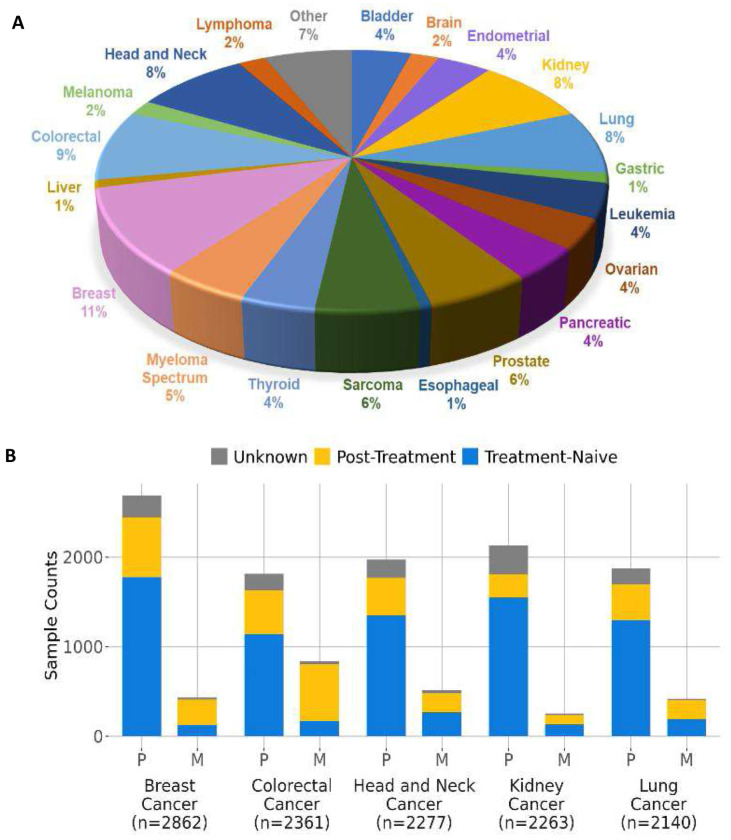
Molecular Data Availability in Avatar. **A.** Pie chart showing disease type distribution (N = 32,557 Avatar patients, version 24.01). **B.** Stacked bar plot showing the number of tumor samples for the top 5 diseases with whole exome and/or whole transcriptome sequencing data available. The counts are broken out to indicate specimens collected from the primary (P) site or a metastasis (M) lesion, with consideration for the timing of specimen collection relative to treatments the patient experienced, post-treatment representing a specimen collected after receiving either radiation, chemotherapy, immunotherapy, a non-corticosteroid hormonal therapy, or any other cancer-related medications. For some patients, multiple biospecimens may have been collected along their medical journey, thus the total number of individual Avatar patients are listed below each disease type, defined as a patient with at least one tumor biospecimen collected and sequenced, and associated clinical data available. Samples labeled as “Unknown” status indicate that the Avatar is missing necessary date information (e.g., per HIPPA regulations, precise age information is suppressed for all Avatars >90 years old); Sample counts as of v24.01.

Unless noted, the Avatar clinicogenomics dataset summarized herein reflects version 25.01 comprising data from 32,557 patients enrolled at the time of the dataset freeze with over 37,000 tumors sequenced. **Supplemental Table 1** provides a comparison of the race and ethnicity representation in the Avatar and The Cancer Genome Atlas (TCGA) datasets,^14,15^ showing a two-seven fold increase in the number of various underrepresented populations within Avatar. The dataset encompasses over 30 cancer types (Figure 2A), with the top five most represented cancer types being breast (11%), colorectal (9%), head and neck (8%), kidney (8%), and lung (8%). The Avatar dataset also includes patients with rare tumors from varying tissue origins (such as ampullary, appendiceal, penile, vulvar, anal, thymic, adrenal gland, and heart) and rare histologies (such as nodular melanoma, pineoblastoma, ependymoma, astrocytoma, oligodendroglioma, meningioma, Ewing’s sarcoma, fibrosarcoma, neuroendocrine tumors, and mesothelioma) that combined comprise 6% of Avatar cases.

Avatar prioritizes patients at high risk of disease recurrence or progression, though it includes all cancer stages and types. It is particularly enriched with data on advanced cancers. Avatar is unique in that sequenced specimens are collected from either the primary tumor (84%) or metastatic sites (16%), with 4% of patients having paired primary/met samples. Furthermore, the resource includes tumors that were treatment-naive (55%) or collected after exposure to radiation, chemotherapy, immunotherapy, or other targeted therapies (35%; remaining ~10% cannot be determined due to masked timepoints after age 90 or unknown from the records). Figure 2B characterizes the distribution of sequenced tumors for the top five cancer types by treatment experience for both primary and metastatic tumor samples.

**Figure 3.**
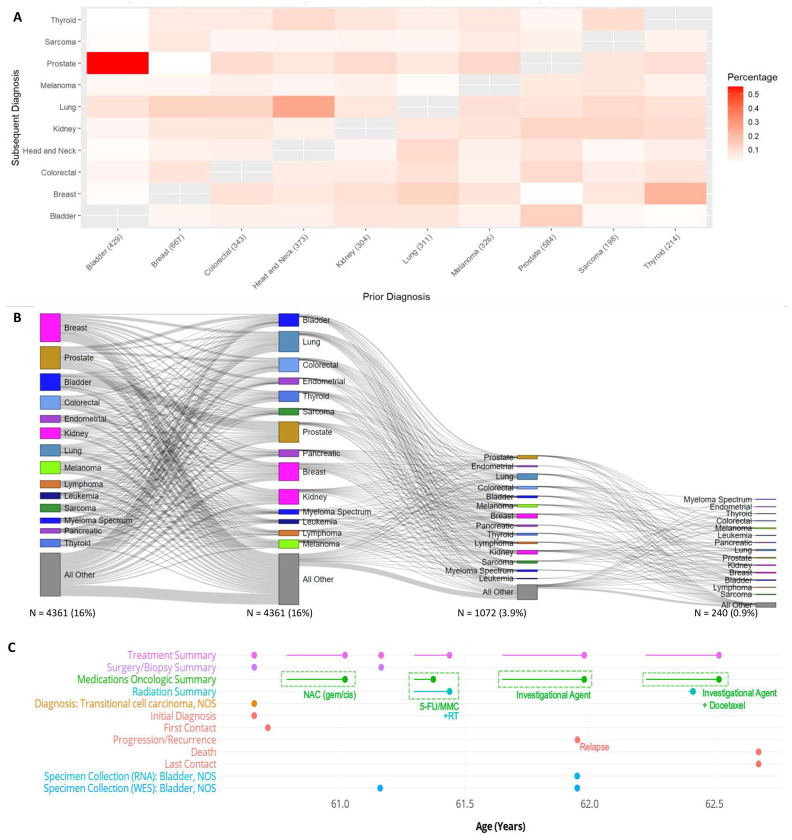
Longitudinal Clinical Data – Capturing the Patient Experience. **A.** Heatmap of propensity for secondary malignancies following initial diagnoses. **B.** Sankey plot of all patients who were diagnosed with multiple disease types showing up to the first 4 diagnoses in the patient’s medical journey. The height of the boxes represents numbers of Avatar patients, and the columns correspond to the order of diagnoses. The gray lines connecting consecutive diagnoses represent the number of patients diagnosed with those subsequent cancer types. The top 14 disease types with the most cases are shown with all other cancer types combined into “All Other.” The number (N) of patients with secondary, tertiary, and quaternary cancers are denoted below. **C.** The medical journey of an individual patient diagnosed with muscle-invasive bladder cancer, with a small number of select clinical data events displayed to show some of the more critical aspects of this patient’s story.

Rich longitudinal clinical data (Figure 1C) enables comprehensive views into patient cancer history. The median follow-up time from first cancer-related contact at an ORIEN institution to last contact or death for all Avatars is 4.7 years (interquartile range: 2.5 to 7.6 years). Such data allows identification of temporal trends in subsequent cancer diagnoses (Figure 3A–B) and detailed individual level treatment patterns (Figure 3C). The frequency and temporal patterns of subsequent cancers can be observed for more than 4361 Avatar patients (16% of total Avatar population) who received multiple diagnoses over time (Figure 3A). Figure 3C provides a visualization of the complex clinical history for an Avatar patient treated for muscle invasive bladder cancer.

### Benchmarking Real World Data Quality and Standardized Molecular Profiling

Avatar reflects real-world clinical practice with a majority of tumors available for sequencing from clinical formalin-fixed paraffin embedded (FFPE) blocks (60% from FFPE and 40% from fresh frozen (FF) tumors). Our RNASeq dataset using a ComBat normalization algorithm per disease cohort and pan-cancer appropriately mitigates non-biological effects on RNA-seq data due to preservation methods. **Supplemental Figure 1** presents breast and colorectal cancer cohorts from Avatar and TCGA in which the FFPE/FF and Avatar/TCGA differences were mitigated and the biological distinctions (i.e., breast vs. colorectal cancer) remained following normalization. These results demonstrate that the Avatar RNA-seq data resource can be compared and/ pooled with other RNA-seq data sources.

**Figure 4.**
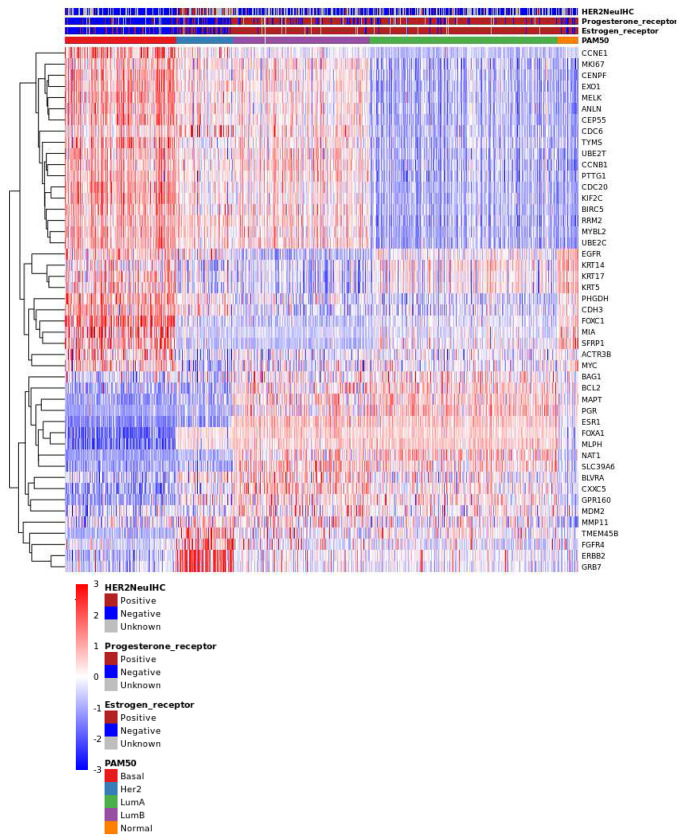
Correlation of IHC Testing and Whole Transcriptome Signatures in Breast Cancer. Heatmap showing breast cancer subtypes as defined by the PAM50 gene signature. The top color bars show results of clinical test by immunohistochemistry (IHC) for HER2, PR, or ER for patients who received this within 6 months of biospecimen collection date.

**Figure 5.**
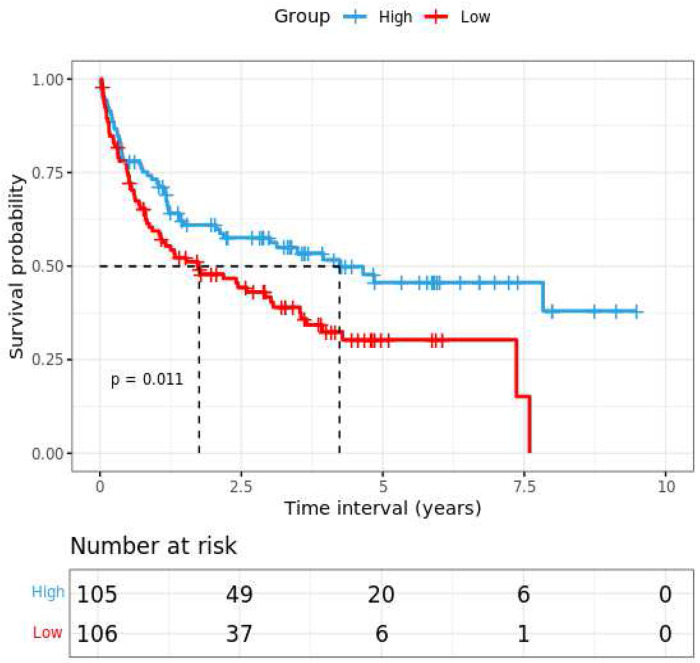
Demonstrating Real World Outcomes Utilizing Avatar. Real-world progression-free survival (rwPFS) for immune-checkpoint-inhibitor-treated melanoma patients, stratified by high (T-cell inflamed) or low (non-T cell-inflamed) gene expression signatures.

To demonstrate the value of transcriptomic data in precision oncology, we generated clinically relevant gene signatures associated with breast cancer subtypes^16,17^ or T-cell inflammation. ^18^ For breast cancer, we examined the association of breast cancer molecular subtypes, as defined by RNA-seq PAM 50 gene signatures,^17^ and clinical biomarker tests (Figure 4). The clinical biomarker test results for ER/PR/HER2 align as expected with the five typical breast cancer molecular subtypes predicted using the PAM 50 gene signature, indicating a good level of confidence in the compatibility of the Avatar clinical and transcriptomic data. Using transcriptomic data, we calculated a summary score for the 18-gene T-cell inflamed signature and dichotomized at the median as “hot” (score above median) or “cold” (score at or below the median).^18^ We examine whether melanoma patients with varying tumor T-cell inflamed gene signatures had different real-world progression free survival (rwPFS) for specific treatment regimens. Melanoma patients with higher expression of the signature, or “hot” tumors, demonstrated significantly longer rwPFS compared to those with “cold” tumors (Figure 5; p=0.011). Cox regression confirmed the prognostic value of the signature summary score as a continuous variable, with a higher score associated with longer rwPFS (**Supplemental Table 2**; Hazard Ratio=0.98 (95% CI 0.97-0.99, p=0.007)), adjusting for age, sex, and site of collection. The observation of longer rwPFS for Avatars with higher expression of the T-cell inflamed signature agrees with the original report of the signature, in which high expression of the signature was associated with a favorable clinical response to immune checkpoint inhibition (ICI) measured by RECIST criteria.^18^

**Figure 6.**
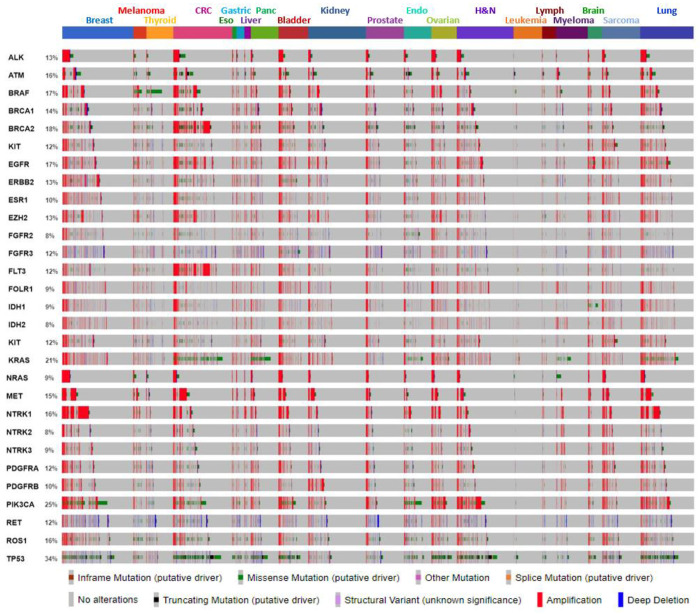
Molecular Profile of Clinically Actionable Biomarkers. OncoPrint from the ORIEN instance of cBioPortal displaying somatic alterations in the top clinically actionable biomarker genes across disease types in Avatar (v24.01). CRC = colorectal; Eso = esophageal; Panc = pancreatic; Endo = endometrial; H&N = head and neck; Lymph = lymphoma. N = 25,518 patients profiled.

To demonstrate insights derivable from the Avatar WES dataset, we show distributions of somatic mutations and copy number alterations in clinically actionable biomarkers with FDA-clear companion diagnostic tests across multiple cancer types (Figure 6), and tumor mutation burden (TMB) scores (**Supplemental Figure 2A**). Overall, both the distribution of somatic mutations and TMB in Avatar replicated previous results from TCGA.^19^ At the tumor site and histology level, we compared somatic mutation rates of known oncogenes and tumor suppressors between lung adenocarcinoma (LUAD; n=891) and lung squamous cell carcinoma (LUSC; n=405). **Supplemental Figure 2B** shows the top six differentially mutated genes, with KRAS (36% in LUAD vs. 4% in LUSC) and TP53 (40% in LUAD vs. 80% in LUSC) mutations showing mutually exclusive profiles (Fisher exact p = 1.8E-8). In a colorectal cancer cohort, we predicted the four-consensus molecular subtyping (CMS) from RNA-seq data^20^ and examined the mutational profiles in the top mutated genes by CMS subtype in **Supplemental Figure 2C**. In addition, Soupir et al. leveraged the Avatar dataset to examine the molecular landscape across a broad group of sarcomas.^21^ In an analysis of 1162 patients with tumor and germline WES, they reported a significantly higher mutation frequency in metastatic tumors compared to primary tumors, which was largely accounted for by an increase in frequency of the most common tumor suppressors TP53 (26% vs. 16%, Chi-squared p = 0.0007) and ATRX (8.9% vs 5.2%, Chi-squared p = 0.04).^21^ These findings in both common and rare cancers demonstrate the broad applicability of this clinicogenomic data resource and the vast potential to generate novel insights into the underpinnings of multiple cancers.

### Translational Research Utilizing Avatar

Representative high impact use cases demonstrate both academic collaborations within ORIEN and collaborations with industry partners to support in-depth discovery and translational research.

#### Immuno-oncology

ORIEN scientists established the immuno-oncology (IO) research interest group (RIG) to identify molecular markers to aid in predicting clinical outcomes, early indications of response or progression, provide biological insight into therapeutic resistance and identify immune-related toxicities.^22,23^ For example, IO RIG scientists utilized real-world clinical and transcriptomic Avatar data from patients with advanced malignancies who underwent immune checkpoint inhibitor treatment to derive an immunoscore based on CD3+ and CD8+ T cell densities. The imputed immunoscore effectively predicted overall survival.^22^ They went on to examine mRNA co-expression levels of PD-1 with 13 immune checkpoints across multiple cancers within the Avatar dataset. They reported co-expression of PD-1 with 13 immune checkpoints and PD-L1 varied across selected malignancies, with cutaneous melanoma and urothelial carcinomas having PD-1 expression correlated with multiple co-inhibitory receptors (cutaneous melanoma: LAG3, TIM3, TIGIT, VISTA and urothelial carcinoma: TIGIT, CTLA4, LAG3, VISTA) and co-stimulatory molecule (cutaneous melanoma: CD137 and urothelial carcinoma: OX40, CD27, CD137, HVEM), while there was limited co-expression observed in pancreatic and ovarian tumors. The IO RIG collaborative multidisciplinary effort represents the synergistic power of a broad partnership represented by the network.

#### Precision Oncology in Multiple Myeloma

In alignment with ORIEN’s founding principles of academic–industry partnership, ORIEN scientists, Aster Insights, and industry collaborators worked together to advance precision oncology for multiple myeloma (MM) patients, a setting where no current guidelines exist to inform optimal therapy selection for individual patients. The team integrated matched Avatar molecular data with *ex vivo* drug sensitivity screening data from the *Ex Vivo* Mathematical Myeloma Advisor (EMMA)^24,25^ in functional genomic analyses to generate transcriptional “footprints” with both predictive and mechanistic utility. These transcriptomic footprints were identified and validated for key MM therapies, including the anti-CD38 monoclonal antibody daratumumab (DARA) and the nuclear export inhibitor selinexor (SELI).^26^ The gene signatures accurately classified clinical responses and demonstrated that optimal disease control occurred with sequential therapy—daratumumab followed by selinexor. This work contributed to the design of a Phase 3 clinical trial (NCT05028348) evaluating selinexor-based therapy following anti-CD38 monoclonal antibody failure. These efforts demonstrate how the Avatar dataset can be leveraged to identify clinically actionable biomarkers and improve therapeutic strategies that directly impact patient care.

#### Computational pipeline to identify the Tumor Microbiome

The tumor microbiome is a relatively understudied aspect of the tumor microenvironment and therefore an ORIEN RIG was established to focus on leveraging Avatar data to explore the tumor microbiome. The Microbiome RIG applied a) computational pipeline to identify and quantify non-human sequences within tumor transcriptome data^27^ and b) a graph-based neural network approach to characterize relationships between microbes and host gene expression.^28^ They identified associations between tumor microbiome and overall survival, age, BMI, and other features. ^27^ This RIG applied their pipelines to specific research questions across disease groups, including 1) a cohort of melanoma patients treated with immunotherapy, where the performance of machine-learning-based outcome predictions improved when incorporating tumor microbe counts, in addition to gene expression^29^ and 2) a cohort of rectal cancer patients treated with radiation, where the presence of certain microbes was associated with radioresistance.^30^ Critically, these efforts benefited from validation of the presence of microbes in the same tumors and follow-up experiments in preclinical models to generate alternative microbe-directed sequencing, metabolomics, epigenetics, and *in situ* datasets to establish causality.

## DISCUSSION

ORIEN is an alliance of academic cancer centers committed to accelerating cancer research through strategic data sharing and collaboration. Founded in 2014, ORIEN was built on a bold vision to transform the pace, scale, and precision of drug discovery by anticipating future needs for biomarker-driven trials. Over the past decade, ORIEN has fostered a robust culture of collaboration which is evident by the over 200 intermember projects initiated since 2018 and establishment of over 13 RIGs that bring together cross-functional scientists, clinicians, and industry experts. A key differentiator of ORIEN is its operating model, which enables dynamic public-private partnerships—most notably with Aster Insights—to support the network’s mission and sustainability. ORIEN continues to play a vital role in uniting cancer centers, patients, and industry around a shared mission to drive scientific discovery, identify novel predictive and prognostic biomarkers, and improve outcomes for cancer patients.

Together ORIEN and Aster Insights have generated the Avatar clinicogenomics data resource that was intentionally designed to support basic, translational, and clinical research in oncology, as well as the drug development lifecycle for industry partners. The Avatar dataset is a deep characterization of a subset of patients enrolled in the TCC protocol with both tumor and germline sequencing and comprehensive clinical data. Paramount to the success of the Avatar program was the creation of foundational workflows and critical infrastructure that enabled the program to overcome the challenges of harmonizing real-world data, establish pipelines for standardized sequencing, and create a structured data model across the cancer centers in ORIEN. Herein, we have demonstrated the value and reliability of the Avatar dataset through our ability to recapitulate well-known findings from other large datasets, such as TCGA^14^, or those generated through large clinical trials. Furthermore, we have provided evidence that the Avatar transcriptomic data can be pooled with external datasets, such as TCGA, increasing the impact of this resource for large scale projects that may require multiple sources, such as when studying the genomics of rare cancers.

Avatar supports a diverse array of studies across over 200 ORIEN-wide scientific publications and is extensively utilized in both industry and academic fields (https://www.asterinsights.com/research/publications). Herein, we highlight several ways that transdisciplinary research groups have leveraged the clinicogenomic data resource to address a broad spectrum of discovery and precision oncology research questions including identification of molecular predictors of treatment response, conducting a molecular landscape analysis across a broad group of rare cancers, resistance mechanisms across tumor types, integrated functional genomics analysis of drug sensitivity, and the application of novel computational pipelines. Since its inception, the ORIEN TCC and Avatar datasets have impacted clinical care. One of the most notable successes of industry use of TCC data was demonstrated in 2019 with Merck’s exploration of PD-L1 expression across >25 tumor types to identify additional indications that warranted prioritization for clinical development of pembrolizumab as a monotherapy beyond melanoma and non-small cell lung cancer, including MSI-high colorectal, head and neck, bladder, triple-negative breast cancers, and gastric cancer.^31^ In collaboration with Celgene, a study leveraging TCC patients refuted the linkage between the use of lenalidomide for treating MM and development of secondary malignancies.^32^ Furthermore, a novel gene expression signature that demonstrated sensitivity to venetoclax in MM tumors with a t(11;4) was developed using Avatar transcriptomic data.^33^ These findings contributed to the design of an ongoing Phase I study (NCT03314181) evaluating venetoclax in combination with daratumumab and dexamethasone (VenDd) in patients with relapsed or refractory MM and the t(11;14) translocation.^34^ We anticipate continued discoveries as the Avatar dataset and our partnerships with industry. These examples highlight the depth and breadth of collaborative, data-enabled research powered by the Avatar dataset and underscore the enduring impact of the TCC patient community in advancing precision oncology and discovery research.

It is crucial to contextualize ORIEN and the Avatar precision oncology clinicogenomic data resource alongside other oncology networks and datasets. ORIEN builds on the standardized sequencing approach pioneered by TCGA^14,35^ and has surpassed its size with over 37,000 sequenced tumors—more than 2.5 times the size of TCGA - and continuing to grow. A key distinction is that TCGA primarily includes sequencing of treatment-naïve, primary tumor specimens, whereas the Avatar dataset comprises a broader range of primary and metastatic samples, collected both before and after treatment exposure. It also features deeper clinical annotation than TCGA,^15^ including longitudinal data on outcomes, disease progression by line of therapy, and contemporary treatments such as immunotherapies and antibody-drug conjugates. Avatar is built on a biobanking framework that allows researchers to request additional biospecimens or analytes, which TCGA does not offer. In terms of patient diversity, Avatar includes a racial and ethnic distribution comparable to TCGA, with an increase in Hispanic/Latino representation and more complete capture of demographic data. Other precision oncology efforts, such as American Association for Cancer Research Genomics Evidence Neoplasia Information Exchange (AACR GENIE)^8^ and Memorial Sloan Kettering-Integrated Mutation Profiling of Actionable Cancer Targets (MSK-IMPACT),^36^ are based on targeted panels used in clinical care and typically cover 300–650 cancer-related genes. In contrast, Avatar includes tumor/normal sequencing data for ~20,000 genes, enabling discovery of novel targets and better understanding of understudied genes involved in tumorigenesis, resistance, and progression. Commercial platforms like Tempus^37,38^ and Caris^9^ also provide sequencing and clinical data for research use, but they often lack the longitudinal depth and biospecimen accessibility available through Avatar. Importantly, ORIEN is the only major effort built on a prospective protocol, which includes patient consent for longitudinal follow-up, data sharing, and recontact. This integrated, patient-centric model positions Avatar as a uniquely comprehensive and flexible resource for advancing precision oncology research.

ORIEN is an independent network that was established primarily by institutional investments from member cancer centers and private funding from Aster Insights. The network has developed a robust infrastructure and culture that enables centralized data sharing across institutions, facilitating both intermember and external collaborations. While not subject to NIH data sharing mandates, ORIEN and Aster Insights have developed a data repository guided by FAIR principles of being Findable, Accessible, Interoperable, and Reusable.^39^ This framework incorporates mechanisms for sharing processed datasets, metadata, and analytic code to enhance reproducibility and transparency in research publications. It also provides controlled access to raw data for peer review when deemed necessary. A project request portal (https://www.oriencancer.org/research-programs) facilitates collaboration requests and supports the engagement of the external community in the shared use of this resource. Although ORIEN’s structure and funding model differ from NIH-supported consortia, the ORIEN data repository aligns with the objectives of contemporary data sharing policies and presents a compelling model for supporting collaborative academic oncology research while providing commercial opportunities for data licensing, generating analytic insights, and other solutions for industry partners in order to continually enhance the network’s data, capabilities, and sustainability.

This scientific collective is a resource of centers dedicated to continuously building and enhancing an integrated, real-time clinical and molecular data ecosystem across its member institutions. In 2023, ORIEN and Aster Insights launched the “Galaxy” initiative as a data resource enhancement focused on automating clinical data feeds and incorporating clinical sequencing and other CLIA-certified diagnostic testing results for as many TCC and Avatar patients as possible. Galaxy is a major step towards the timely and efficient identification of patients who are optimally suited for a specific treatment or clinical trial based on their clinical and molecular profile, enabling clinical trial matching via the ORIEN Clinical Trial Network (OCTN) which has been a foundational goal of the network since its origin. Other efforts to improve resources and infrastructure are also well underway, including the alignment of all data models with Observational Medical Outcomes Partnership (OMOP) standards^40^ to facilitate broader data harmonization, expansion into new modalities such as digital pathology with whole slide images (WSI), and the development of cloud-based research workbenches to enable secure, collaborative analysis of shared datasets without local downloads.

Precision oncology data resources have tremendous potential to leverage the power of artificial intelligence (AI) and machine learning.^41^ Zephyr AI (https://www.zephyrai.bio) is Aster Insights’ new partnering parent company and contributes a robust suite of AI and machine learning tools that expand the translational potential of the ORIEN network. Zephyr’s proprietary platforms and AI products offer a wide range of innovative tools to advance precision oncology. These include the Nexus^™^ platform, which transforms complex real-world data (RWD) into real-world evidence to support rapid cohort discovery, outcomes analysis, predictive modeling and validation; and the AIM-x^™^ suite - a collection of multi-modal, interpretable AI models for drug response prediction and expression reconstruction. All AIM models are designed to operate on clinical and molecular data inputs routinely collected in real-world settings and can be fine-tuned to partner-specific therapies or commercial LDT assays to enable the co-development of AI-enabled companion diagnostics (AI-CDx^™^). The integration of Zephyr’s scalable, explainable AI with ORIEN’s longitudinal data, network of expert clinical investigators, and trial infrastructure has the potential to unlock powerful new capabilities for prospective and retrospective validation, fit-for-purpose cohort construction, and in silico clinical trial enrichment. Together, these resources provide the foundation for the ORIEN Clinical Trial Network to become a leading partner for precision oncology development and advancing the long-standing goal of delivering point-of-care tools that translate decades of research and real-world outcomes into individualized patient insights.

ORIEN remains committed to growing the network and TCC to advance precision medicine, drive scientific discovery, and promote collaboration. With evolving capabilities toward real-time data integration, expanded access to AI capabilities in collaboration with Zephyr AI, and a growing ecosystem of interoperable platforms aligned with FAIR principles, the Galaxy and Avatar resources are uniquely positioned to enable discovery, power clinical trial innovation, and honor the contributions of patients by driving forward meaningful, high-impact cancer research.

## ONLINE METHODS

### Participant Enrollment in Total Cancer Care

All ORIEN alliance members utilize a standard Total Cancer Care (TCC) protocol ^6,7^ or biospecimen collection protocol that share common core elements within the TCC protocol. All participants read and sign an IRB-approved Informed Consent Form to allow collection of clinical data and specimens from routine medical care, storage of biospecimens for long-term use, lifetime follow-up unless consent is withdrawn, contact for future research studies when appropriate, and research data to be shared with multiple stakeholders to advance cancer knowledge. New patients are continually enrolled to TCC, while others may withdraw from network data sharing upon their request. All biospecimens collected as part of TCC reside within the ORIEN members biorepository or pathology department until requested for a project.

### ORIEN Avatar Project

TCC consented patients with biospecimens, and who meet eligibility criteria, may be included into the Avatar Project. Avatar includes research use only (RUO) grade whole-exome tumor and germline sequencing, transcriptome RNA sequencing, and collection of deep longitudinal clinical data with lifetime follow up. The Avatar dataset is updated continuously with batched releases every 2-3 months to add additional patients and update data on existing patients. The version 25.05 Avatar release files were used to generate the figures in this snapshot of the ever-evolving dataset.

### Sequencing Methods (RUO)

At each ORIEN site, solid tumor samples from TCC consented patients eligible for Avatar are reviewed by a clinical pathologist. Solid tumors are required to be > 30% tumor; if not, macrodissection is performed to increase tumor content. Avatar specimens undergo nucleic acid extraction and sequencing at HudsonAlpha (Huntsville, AL), Fulgent Genetics (Temple City, CA), or Azenta Life Sciences (South Plainfield, NJ). For frozen and OCT tissue DNA extraction, Qiagen QIASymphony DNA purification is performed, generating 213 bp average insert size. For frozen and OCT tissue RNA extraction, Qiagen RNeasy Plus Mini kit is performed, generating 216 bp average insert size. For FFPE tissue, Covaris Ultrasonication FFPE DNA/RNA kit is utilized to extract both DNA and RNA, generating 165b bp average insert size. For DNA sequencing, preparation of Aster Insights Whole Exome Sequencing (WES) libraries involves hybrid capture historically using an enhanced Roche NimbleGen (Madison, WI; 34.7 Mb) or IDT WES kit (Coralville, IA; 38.7 Mb) with additional custom designed probes for double coverage of 440 cancer genes and most recently updated to an expanded Twist Biosciences probe set (San Francisco, CA; 44.8 Mb) designed for expanded coverage of specified intronic regions and double coverage of 620 cancer-related genes. Library hybridization is performed at either single or 8-plex and sequenced on an Illumina NovaSeq 6000 and NovaSeq X instrument generating a minimum of 100 bp paired reads. WES is performed on tumor/normal matched samples with the normal covered at 100X and the tumor covered at 300X (additional 440 or 620 cancer genes covered at double coverage; 200X for normal and 600X for tumor). Both tumor/normal concordance and gender identity QC checks are performed. Minimum threshold for hybrid selection is >80% of bases with >100X fold coverage for tumor and >50X fold coverage for normal. RNA sequencing (RNA-Seq) is performed using the Illumina TruSeq RNA Exome with single library hybridization, cDNA synthesis, library preparation, sequencing a minimum of 100 bp to a coverage of 100M total reads / 50M paired reads. Gene expression was quantified as Transcript Per Million (TPM), log2(TPM+1) transformed, and ComBat normalized to adjust for batch effects related to preservation method.

### Whole Exome and Transcriptome Sequencing Analysis Pipelines

Matched tumor and normal WES adapter-trimmed FASTQ files are aligned to the human genome reference (GRCh38/hg38) using the BWA-MEM aligner (sentieon_release_201911). Resulting alignment files are sorted, duplicate reads are marked, and base qualities recalibrated using the Sentieon driver to calculate QC metrics. Alignment files are compressed in .cram format, which can be converted to .bam format using SAMtools. Haplotyper is used to detect single nucleotide variants (SNVs) and insertions/deletions (INDELs) for both germline and tumor samples, independently. For tumor/normal pairs, TNhaplotyper2 is used for detection of somatic SNPs and INDELs with the –trim_soft_clip option enabled. Variant files are provided in compressed Variant Call Format (.vcf.gz) and Genomic VCF (.g.vcf.gz) file formats. Variant annotation is performed using Funcotator (GATK v4.1.6.0). Functional annotation includes amino acid coding changes, association with the Catalogue of Somatic Mutations in Cancer (COSMIC), ClinVar relationships among human variants and phenotypes, the Genome Aggregation Database (gnomAD) of population polymorphisms, and familial cancer genes. A Panel of Normals (PoN) filter is applied on Funcotator annotated tumor and somatic vcf. Mutations are filtered against the PoN mutation list, adding “panel_of_normals” in FILTER field. The PoN filter tag is used to reduce the False Discovery Rate (FDR) for somatic mutations by identifying both population polymorphisms and systematic sequencing artifacts. The PoN includes mutations that are present in >0.5% of the entire ORIEN Avatar population of unrelated germline samples.

For RNA-sequencing data, adapter sequences are trimmed from the raw tumor sequencing FASTQ file. Adapter-trimming via k-mer matching is performed along with quality-trimming and filtering, contaminant-filtering, sequence masking, GC-filtering, length filtering and entropy-filtering. The trimmed FASTQ file is used as input to the read alignment process. The tumor adapter-trimmed FASTQ file is aligned to the human genome reference (GRCh38/hg38) and the Gencode genome annotation v32 using the STAR aligner. The STAR aligner generates multiple output files used for Gene Fusion Prediction and Gene Expression Analysis. STAR-Fusion (v1.8.0) and Arriba (v1.1.0) gene fusion algorithms are applied to the STAR aligner output files. Gene Fusion predictions from both STAR-Fusion and Arriba are merged into a single output file that removes duplicate putative gene fusion calls, removes putative gene fusion calls of low confidence – reporting gene fusions with at least one (1) junction read and at least one (1) spanning read, and removes gene fusion calls occurring within the same gene, within snoRNAs, within rRNAs, or mitochondrial genes.

### Clinical Data Abstraction, Harmonization, and Standardization

Data abstractors collect data from the medical records of TCC patients into a standard ORIEN-wide Avatar case report form set through facility-specific REDCap electronic data collection (EDC) tool following consistent Avatar abstraction guidelines. Medical record data abstraction is the primary source of data across participating member institutions, followed by extraction of data from the EMR or institutional data warehouses. Clinical data curated for the Avatar program include demographics, self-reported clinical history and risk factor data, cancer diagnoses and staging, comorbidities, prior treatments and procedures, performance status, laboratory and radiologic test results, toxicities, outcomes, and survival status (Figure 1C). Treatment response, progression-free survival, disease-specific survival, and overall survival are derived from active follow-up of all patients. Avatar patients have a baseline set of data collected at the time the tumors are shipped for sequencing and updates submitted in 6-month intervals. A limited-dataset is securely transferred to Aster Insights, who harmonizes abstracted clinical data elements and molecular sequencing files into a standardized, structured format to enable the sharing of aggregated de-identified data across the Network.

The Clinical Data Management Team at Aster Insights conducts bi-monthly training sessions for ORIEN abstraction staff. These training sessions provide updates to abstraction guidance, diagnosis and staging references, and various other oncology topics. Case review examples are also provided, based on Member-submitted questions and requests. Aster Insights has worked with ORIEN Members to establish Data Monitoring Services, providing case review and updates for select case lists. These monitoring activities provide insight into Member abstraction staff practices and capabilities, culminating in detailed reports comparing site-abstracted data to the Aster Insights source verification, with any deviations noted. These reports are used to establish conformity with best practices or to recommend additional training opportunities for the Member’s clinical abstraction staff involved.

### Data Quality Processes and Measures

Upon ingestion, the Avatar clinical data records are subjected to more than 150 automated quality checks that evaluate date inconsistencies, domain-level concordance with populated records, and determine critical field population. Manual reviews are conducted to validate the biospecimen metadata alignment with the clinical data with in-depth review of select record sets. Cumulative discrepancy reports are issued to the submitting ORIEN Member for the review, remediation, and resubmission of a corrected data set, and the updated data are again quality checked. Additionally, the cumulatively collected and updated clinical data are subjected to periodic retrospective consistency and completeness reviews. The retrospective review will re-evaluate all clinical data collected, identify potential conflicting records and evaluate the completeness of all clinical data collected. For patients confirmed to be deceased, we require all clinical domains being verified after the date of death to assure all clinical data available were reported. For patients without death information, we require all clinical domains to be verified within the past 12 months. A detailed work list was generated including all patients that have death date conflict or incomplete clinical data reports, and returned to ORIEN member sites to correct any mistakes and make sure complete clinical data are abstracted.

### Real-World Progression-Free Survival Analysis and Median Follow-up

A cohort of 211 Avatar melanoma patients was identified with annotated diagnosis dates, evaluable rwPFS endpoints and with an initiation date of immune-checkpoint inhibitor (ICI) therapy after RNA-seq specimen collection. Median follow-up was calculated according to the reverse Kaplan-Meier.^42^ Progression events were defined as: annotated progression/recurrence in clinical records, annotation of therapy stopped due to progression, identification of new metastases, or death, with right censorship at date of last contact for patients without a progression event. The summary score for the gene T-cell inflamed signature ^18^ is a linear combination of normalized, log2 Transcript Per Million (TPM) values for the 18 reported genes, using simple weights of 1 and −1 for genes positively and negatively associated with clinical response, respectively, in the original report.

## Supplementary Material

This is a list of supplementary files associated with this preprint. Click to download.

• SupplementaryDataORIENFINAL07012025.docx

## Data Availability

The data used in this study was generated through private funding by Aster Insights (www.asterinsights.com) in collaboration with the Oncology Research Information Exchange Network (ORIEN, www.oriencancer.org). Inquiries regarding access to the data or collaboration within ORIEN should be submitted to the corresponding author or can be submitted here at https://researchdatarequest.orienavatar.com/ .
